# Role of Exosomes During Mycobacterium Tuberculosis Infection: A Double-Edged Sword

**DOI:** 10.3390/biomedicines14091972

**Published:** 2026-09-01

**Authors:** Varsha Rawat, Vinod Yadav, Abhishek Mishra

**Affiliations:** 1 Department of Pathology and Genomic Medicine, Houston Methodist Research Institute, Houston, TX 77030, USA; vrawat@houstonmethodist.org; 2 Department of Microbiology, Central University of Haryana, Mahendragarh 123031, Haryana, India; vinodyadav@cuh.ac.in

**Keywords:** *Mycobacterium tuberculosis*, exosomes, macrophages, immune modulation

## Abstract

Exosomes are nanoscale, double-membrane extracellular vesicles of endocytic origin that are released by diverse cell types under both physiological and pathological conditions. They have emerged as critical mediators of intercellular communication and immune regulation during homeostasis and infection. Accumulating evidence indicates that pathogen-infected mammalian cells actively secrete exosomes enriched with host- and pathogen-derived components, including proteins, lipids, DNA, and regulatory RNAs such as microRNA (miRNA) and circular RNA (circRNA). In the context of infection, exosomes exhibit dual and often opposing functions, contributing to both host defense and disease progression. *Mycobacterium tuberculosis* (*Mtb*), the causative agent of tuberculosis and a leading cause of mortality from infectious diseases worldwide, has been shown to modulate exosome biogenesis and alter cargo composition in exosomes when secreted by macrophages. These exosomes carry mycobacterial antigens and immunomodulatory molecules that can influence host immune responses, thereby shaping disease outcomes. This review summarizes current insights into the biogenesis and functional roles of exosomes during *Mtb* infection, highlighting their contributions to host–pathogen interactions, immune modulation, and disease pathogenesis. We further discuss the potential of exosomes as biomarkers and therapeutic targets in tuberculosis, emphasizing their dual roles in mediating protective and detrimental effects.

## 1. Introduction

*Mycobacterium tuberculosis* (*Mtb*) is an intracellular pathogen that primarily infects the lungs following inhalation of aerosolized droplets released by infected individuals. According to the World Health Organization (WHO) Global Tuberculosis Report (2025), an estimated 10.7 million people developed tuberculosis, and approximately 1.23 million deaths were attributed to the disease, underscoring its status as one of the leading causes of mortality from infectious diseases worldwide [1]. In the lungs, *Mtb* is phagocytosed by alveolar macrophages, initiating a localized pro-inflammatory response. This leads to the recruitment of immune cells, including monocytes, macrophages, and neutrophils, to the site of infection, resulting in the formation of granulomas, which are organized cellular structures and a hallmark of tuberculosis. Within granulomas, macrophages differentiate into specialized forms such as multinucleated giant cells, foamy, and epithelioid macrophages. As the adaptive immune response develops, lymphocytes, particularly CD4^+^ T cells, accumulate and contribute to the structural organization and functional dynamics of the granuloma. Protective immunity against active tuberculosis is largely mediated by cellular immune responses, with CD4^+^ T cells and macrophages playing central roles in host–pathogen interactions [2]. Emerging evidence suggests that exosomes derived from *Mtb*-infected cells contribute to this process by modulating immune signaling and influencing granuloma formation and function, thereby impacting disease progression [3,4].

Exosomes are secretory extracellular bioactive nanovesicles, 30–150 nm in size, with a lipid bilayer of endocytic origin, released by nearly all human cells under both normal and pathological conditions, and play a key role in maintaining dynamic homeostasis [5]. These vesicles are released into the extracellular milieu through the fusion of multivesicular bodies with the plasma membrane and function as mediators of intercellular communication, acting as paracrine or endocrine signaling effectors that transfer information from donor to recipient cells. The composition of exosomes reflects their cellular source, containing selective transcriptome (mRNA, miRNA, lncRNA, and circRNA), proteome, and lipidome profiles [6,7]. They can be isolated from a variety of body fluids, including blood, urine, saliva, plasma, breast milk, semen, amniotic fluid, malignant ascites, synovial fluid, and cerebrospinal fluid [8,9,10,11]. The cargo encapsulated within exosomes, comprising specific cellular proteins, signaling peptides, nucleic acids, and lipids, is enclosed within a membrane that protects it from RNases and proteases. A wide range of cells can secrete exosomes, including platelets, mesenchymal stem cells, B cells, T cells, dendritic cells, neurons, tumor cells, and epithelial cells, and these vesicles remain stable in body fluids for extended periods [12,13,14,15,16]. Recent studies have highlighted the potential of exosomes in biomarker discovery and the diagnosis of tuberculosis [17].

**Figure 1:**
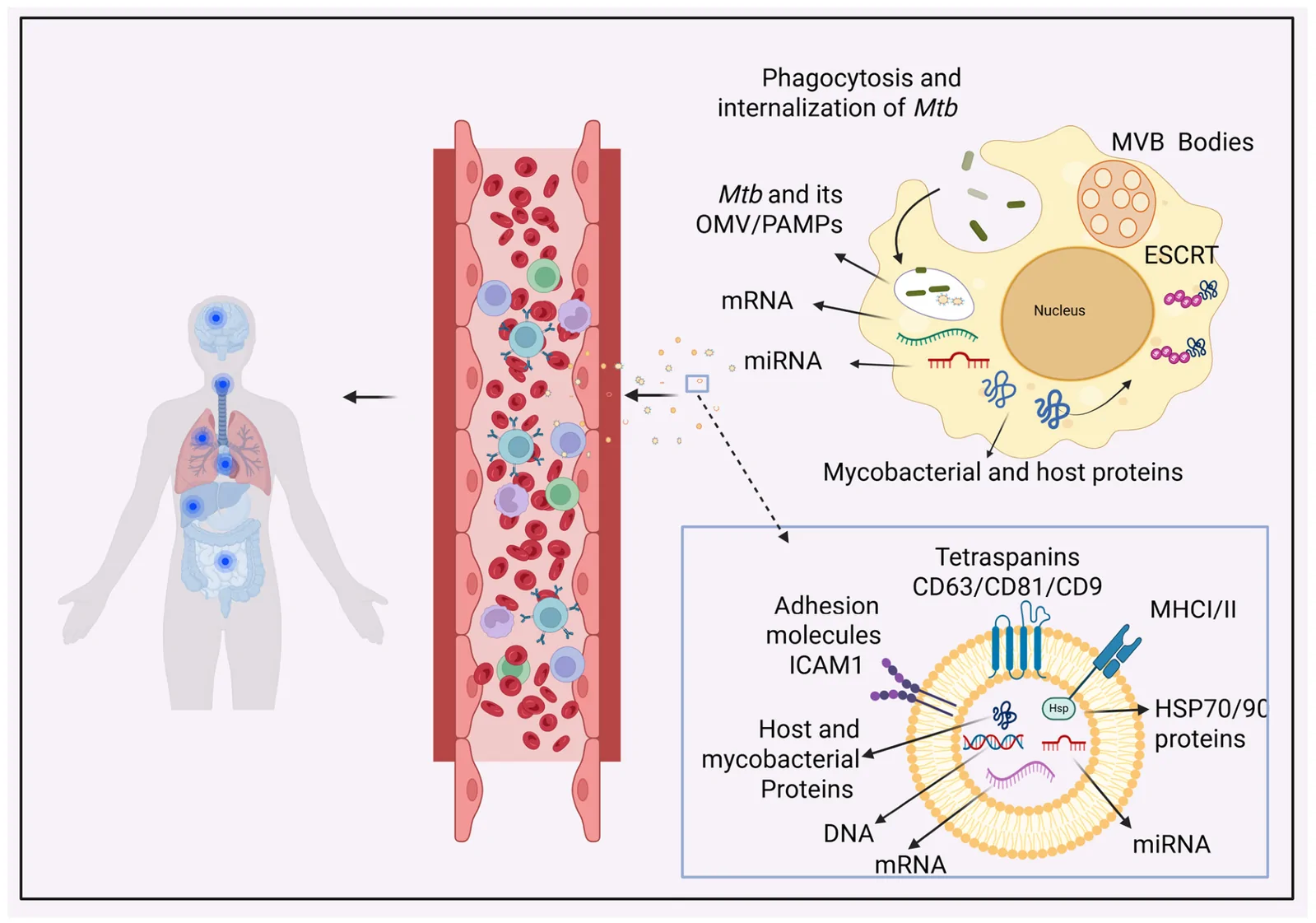
Immunomodulatory crosstalk between *Mtb*-infected macrophages and exosome-mediated communication. Following phagocytic uptake, *Mtb* inhibits phagosome–lysosome fusion and secretes virulence factors into the host cytoplasm. Host- and bacterial-derived proteins, lipids, mRNAs, and non-coding RNAs are selectively packaged into exosomes via the ESCRT pathway, incorporated into multivesicular bodies (MVBs), and released to modulate neighboring and distant cells. Exosomes derived from *Mtb*-infected macrophages contain host and bacterial proteins, lipids, mRNAs, and small RNAs, together with characteristic exosomal markers including CD63, CD81, TSG101, ICAM, and MHC class II molecules. After entering the circulation, these exosomes disseminate systemically, regulate immune responses in distal organs, and carry molecular signatures with potential diagnostic and prognostic value for tuberculosis.

Exosomes are a major source of circulatory RNA in body fluids, as these vesicles participate in horizontal gene transfer by trafficking exosome shuttle RNA (esRNA), most commonly mRNA and miRNA, from donor or parent cells to recipient cells [14,15,18]. Uptake occurs via receptor-mediated endocytosis, membrane fusion, or other internalization pathways, allowing the exosomal RNA-cargo together with associated effector proteins to modulate gene expression in the recipient cells (Figure 1). This process can induce specific genetic and epigenetic changes, often resulting in functional outcomes such as post-transcriptional gene silencing, either upregulated or downregulated [19].

In this review, we summarize recent advances in understanding the role of exosomes derived from *Mtb* infection in modulating host epigenetic mechanisms and their impact on disease progression. We further evaluate the production and functional activity of these exosomes during *Mtb* infection and discuss whether they confer protective benefits to the host immune system or facilitate pathogen survival.

### 1.1. Literature Search Strategy

A comprehensive literature search was performed to identify studies investigating the role of exosomes and extracellular vesicles in *Mtb* infection. Electronic databases, including PubMed, Scopus, Web of Science, and Google Scholar, were searched for articles published up to July 2026. Search terms included combinations of “tuberculosis,” “Mycobacterium tuberculosis,” “exosomes,” “extracellular vesicles,” “membrane vesicles,” “extracellular RNA,” “microRNA,” “long non-coding RNA,” “circular RNA,” “proteomics,” “lipidomics,” “biomarkers,” “diagnosis,” “vaccines,” and “host-directed therapy.”

Original research articles, systematic reviews, and relevant review articles published in English were considered. Studies focusing on exosome biogenesis, molecular cargo, host–pathogen interactions, immune regulation, diagnostic and prognostic biomarkers, vaccine development, and therapeutic applications in tuberculosis were prioritized. Seminal studies were included to provide historical context, while particular emphasis was placed on recent publications (2020–2026) to ensure that the review reflects the latest advances in the field. References cited within relevant articles were also manually screened to identify additional eligible studies.

### 1.2. Exosome Isolation and Characterization

Several methods have been developed to isolate exosomes, each differing in yield, purity, scalability, and suitability for downstream applications. The gold-standard approach is differential ultracentrifugation, which involves sequential centrifugation steps followed by purification using density gradient centrifugation, typically with a linear sucrose gradient of 1.13–1.19 g/mL [20]. Differential ultracentrifugation remains the most widely used method in TB research for isolating exosomes from infected macrophages and biological fluids, although it is time-consuming and may co-isolate contaminants [21,22]. In addition, chemical precipitation methods are widely used; these rely on reducing exosome solubility to precipitate intact vesicles from biological fluids or cell culture supernatants. Density gradient centrifugation provides higher purity and is preferred for proteomic analyses but is technically demanding [23]. Other commonly used techniques include ultrafiltration and size-exclusion chromatography, offering faster isolation while preserving vesicle integrity; SEC is increasingly used in TB biomarker studies due to its reproducibility. Polymer-based precipitation enables rapid processing of clinical samples but often co-precipitates non-vesicular proteins, limiting its use in functional studies. Immunoaffinity capture provides high specificity by targeting exosomal surface markers (e.g., CD63 and CD81) but is costly and unsuitable for large-scale applications. Emerging microfluidic platforms require minimal sample volumes and show promise for TB diagnostics, although clinical validation is still needed [24]. Standardization of isolation methods following the MISEV2018 guidelines is essential to improve reproducibility across TB studies [23].

Following isolation, exosomes are characterized using complementary analytical methods. Electron microscopy enables visualization of vesicle morphology, while nanoparticle tracking analysis (NTA) provides quantitative measurements of size distribution and concentration. Flow cytometry (FACS) and Western blotting are routinely used to detect canonical exosomal markers, including tetraspanins CD63, CD81, and CD9 as well as proteins such as Tsg101 and Hsc70. Proteins involved in vesicle biogenesis and trafficking, including Alix and LBPA-binding proteins, are also commonly assessed. Immunoaffinity-based methods using magnetic beads further allow the isolation of highly purified exosome populations [21].

The molecular composition of exosomes varies depending on their cellular origin and the physiological or pathological state of the donor cells. They are enriched in a diverse array of biomolecules, including proteins, lipids, carbohydrates, and nucleic acids, reflecting their dynamic role in intercellular communication.

### 1.3. Exosomes Are Intercellular Messengers

Initially discovered in the 1980s, exosomes were considered merely as cellular “garbage dumpers,” responsible for the removal of waste and toxins. Johnstone et al. first reported that during reticulocyte maturation, transferrin receptors were eliminated with the help of exosomes [25]. However, research over the past decade has revealed that exosomes are released by diverse cell populations, including immune cells such as T cells, B cells, dendritic cells, and macrophages, where they play key roles in immune modulation, including antigen presentation. A breakthrough in exosome research came when Valadi et al. demonstrated that these vesicles are bioactive and contain RNA molecules, termed exosome shuttle RNA (esRNA) [26]. Notably, the microRNA and mRNA profiles of mast cell-derived exosomes differ from those of other cells, and the RNA cargo is transcriptionally active in recipient cells. Since then, exosomes have been isolated from nearly every cell type, and their roles in disease pathologies have been extensively investigated. Exosomes derived from cancer cells, for example, have been shown to contribute to metastasis, angiogenesis, and tumor progression [27]. Beyond their pathological roles, exosomes have also demonstrated potential as cell-free vaccines, drug delivery vehicles, and biomarkers in infectious diseases [13,28,29,30,31].

Once secreted, exosomes enter the interstitial space and eventually reach the systemic circulation, enabling them to mediate intercellular communication and epigenetic regulation [32]. In addition to transferring bioactive molecules between cells, exosomes can alter the behavior of nearby and distant cells through paracrine and endocrine signaling mechanisms [33]. Studies have demonstrated differential expression of miRNAs in macrophage-derived exosomes within the tuberculosis-infected bone microenvironment, highlighting their role in modulating host immune responses during infection [34]. Furthermore, the unique ability of exosomes to cross the blood–brain barrier underscores their potential involvement in neurological disease progression [35]. Several studies have shown that *Mtb* infection alters the protein and lipid composition of circulating exosomes according to disease state and latent tuberculosis infection treatment status [36,37]. Distinct exosomal signatures have also been identified in patients infected with non-tuberculous mycobacteria and *Mtb* [38]. Collectively, these findings indicate that exosomes can exert diverse effects depending on the stage and state of tuberculosis, influencing not only local cellular environments but also distant organs throughout the body.

### 1.4. Extracellular Vesicles—Exosomes and Outer Membrane Vesicles

Human cells release subpopulations of shedding vesicles categorized based on their biogenesis, composition and size. These vesicles are chiefly apoptotic bodies, microvesicles and exosomes. Apoptotic bodies rise from dead or apoptotic cells while microvesicles arise from the budding of plasma membrane and range from 30 to 2000 nm in size. Among all the shedded vesicles, exosomes are the tiniest ones, varying between 30 and 150 nm in size, and have an endo-lysosomal origin containing a selective sorted cargo of proteins, lipids and RNAs [39,40].

Analogous to human cells, bacteria also communicate with each other by producing outer membrane vesicles (Gram-negative) and membrane vesicles (Gram-positive) containing the same components as the parent bacterium, including most virulent proteins, genetic material, toxins, DNA and RNA, and are similar to exosomes in biophysical aspects [41,42]. The extracellular bacterium maintains quorum sensing and promotes dynamic homeostasis through OMVs [43]. OMVs are also considered as sample sacks with a 50–250 nm sized lipid bilayer. These vesicles were first discovered and characterized in *N. meningitidis* and *V. cholera* during natural growth in vitro [44]. Thus, OMVs contain identical biological material as found in the parent bacterium but in non-replicative form, and they are also involved in bacterial pathogenesis, disease progression, inflammation and immune modulation [45]. However, the role of these vesicles is poorly understood in the context of intracellular pathogen infection, particularly in *Mtb* infection and how these vesicles are transported outside of the infected host cell. More recently, the dynamin-like proteins IniA and IniC have been identified as key regulators of OMV biogenesis [46]. Mycobacterial OMVs also contribute to tuberculosis pathogenesis. *Mtb*-derived OMVs exacerbate infection and lung pathology in mice [47]. Subsequently, studies showed that subcutaneous administration of mycobacterial EVs, alone or as an adjunct to BCG vaccination, may serve as an effective vaccine strategy [47]. Mycobacterial OMVs contain immunogenic lipids and proteins that can effectively prime immune responses; however, their application as vaccines has been limited by poor stability and heterogeneous size. To overcome these limitations, OMV-coated gold nanoparticles (OMV-AuNPs) have been developed and shown to enhance macrophage and dendritic cell activation through TLR2- and TLR4-mediated pathways [48]. OMVs have also emerged as potential natural antitubercular drug delivery vehicles by improving intracellular drug accumulation [49]. Recent studies have highlighted the therapeutic and vaccine potential of mycobacterial OMVs. BCG-derived outer membrane vesicles (B-OMVs) induce trained immunity and protect against experimental polymicrobial sepsis with minimal toxicity, making them a promising alternative to conventional BCG vaccines and *E. coli*-derived OMVs. Mechanistically, B-OMVs promote hematopoietic stem cell expansion and myelopoiesis through TLR2-dependent activation of aerobic glycolysis and epigenetic reprogramming, leading to enhanced macrophage phagocytic activity [50].

It can be hypothesized that *Mtb* takes advantage of the human vesicle trafficking system and delivers most of its antigenic peptides and effector molecules in the exosome vesicular transport system and maintains pro-inflammatory states. During infection, both kinds of extracellular vesicles are produced and can be either pathogen- or host-cell-derived [51]. Overall, bacterial EVs carry pathogen-associated molecular patterns and antigens that simultaneously activate innate and adaptive immunity, highlighting their dual role in disease progression and their promise as diagnostic, therapeutic, and vaccine platforms.

### 1.5. Exosomes in Mtb Infection

As an intracellular pathogen, *Mtb* exploits the host vesicular trafficking system to modulate immune responses and enhance its survival [52]. *Mtb*-derived components are released within host-cell-derived exosomes, despite the pathogen being confined within phagosomes of infected macrophages. These exosomes enable communication with the extracellular environment and influence uninfected bystander cells by delivering both host- and pathogen-derived molecules, thereby modulating immune surveillance and promoting immune dysregulation.

**Figure 2:**
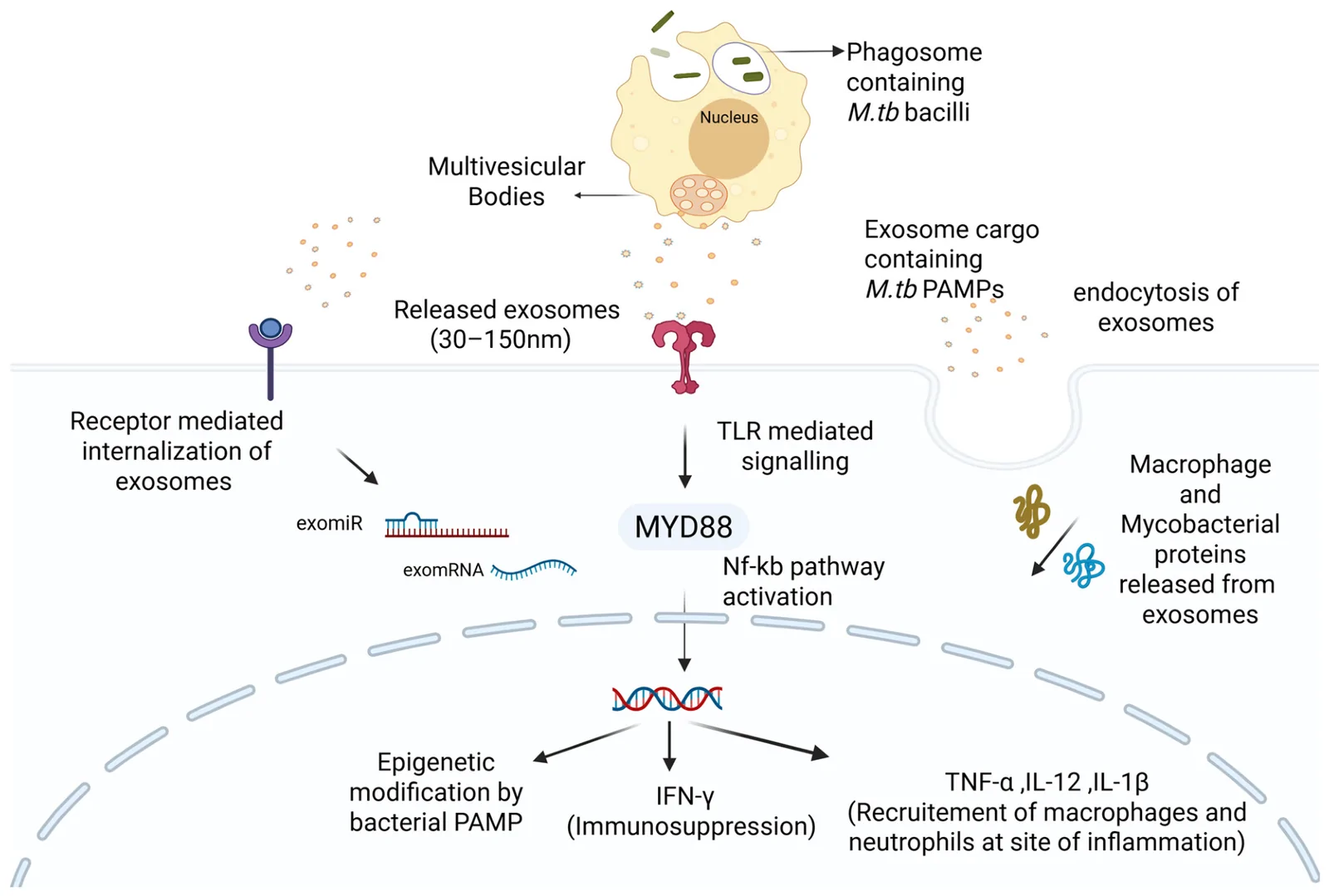
Reprogramming of bystander cells by macrophage *Mtb*-derived exosomes.

Exosomes released from macrophages infected with *Mtb* or *M. bovis* have been shown to contain pathogen-associated molecular patterns (PAMPs) that modulate immune responses via Toll-like receptor (TLR) and MyD88-dependent pathways (Figure 2). For instance, exosomes isolated from bronchoalveolar lavage fluid (BALF) of *M. bovis*-infected mice contain lipoarabinomannan (LAM), a 19 kDa mycobacterial cell wall component, which induces the production of TNF-α and IL-12 and promotes recruitment of macrophages and neutrophils, thereby sustaining a pro-inflammatory response [52,53]. Similarly, mycobacterial lipids such as LAM and phosphatidylinositol mannosides (PIMs) are trafficked from phagosomes to multivesicular bodies (MVBs) and subsequently released via extracellular vesicles [54]. During exosome biogenesis, early endosomes mature into late endosomes and MVBs containing intraluminal vesicles (ILVs) (Figure 1). Cargo is sorted into ILVs through ESCRT-dependent or ESCRT-independent mechanisms before MVBs either fuse with the plasma membrane to release exosomes or with lysosomes for degradation [55,56,57]. In *M. tuberculosis* infection, soluble mycobacterial proteins are internalized, ubiquitinated, and subsequently released via exosomes [58]. Furthermore, ESCRT-III is recruited to Mtb-containing phagosomes in an ESX-1-dependent manner, while the mycobacterial virulence factors EsxG and EsxH antagonize ESCRT-mediated repair of damaged endolysosomal membranes, facilitating intracellular survival [59]. Although *Mtb* proteins and lipids are trafficked into host multivesicular bodies and subsequently released in exosomes, the precise molecular mechanisms by which the pathogen manipulates the ESCRT machinery, cargo sorting, and MVB-lysosome fate decisions remain poorly understood. Current evidence suggests that exosome biogenesis involves both ESCRT-dependent and ESCRT-independent pathways, together with Rab GTPases, SNARE proteins, and tetraspanins that regulate MVB trafficking and membrane fusion. Whether *Mtb* directly hijacks these pathways or indirectly remodels endosomal trafficking to favor exosome release requires further investigation.

Our work on tuberculosis vaccine development consistently demonstrates that robust activation of innate immunity is a prerequisite for generating durable protective immune responses. Live attenuated (Δ*fbpA*Δ*sapM*, triple- and quadruple-knockout *M. tuberculosis* strains), viral-vectored (bovine adenovirus–Ag85B), and subunit nanovaccine platforms based on the mycobacterial lipid trehalose 6,6′-dimycolate all elicit protection by enhancing innate immune activation, antigen presentation, and subsequent adaptive immunity [60,61,62,63,64]. These findings support the hypothesis that exosomes carrying *Mtb* antigens, lipids, or other bacterial components may similarly induce trained immunity and potent antigen presentation, making them attractive candidates for next-generation TB vaccines and host-directed immunotherapies. Interestingly, Exosomes enriched with mycobacterial lipid antigens can activate naïve macrophages, inducing the production of TNF-α, RANTES, and iNOS, and thereby promoting inflammation (Figure 2). Additionally, exosomes derived from *M. bovis* BCG-infected macrophages can activate antigen-specific CD4^+^ and CD8^+^ T cells both in vitro and in vivo, highlighting their role as an alternative mechanism of antigen presentation [65]. Exosomes are important mediators of host immunity during *Mtb* infection, serving as carriers of extracellular antigens that promote T-cell activation and antibacterial immune responses [66]. Exosomes released from *Mtb*-infected macrophages induce the differentiation of naïve monocytes into functionally active macrophages through MAPK/MK2- and NF-κB-dependent signaling, a response distinct from that elicited by exosomes from uninfected macrophages. *Mtb* infection also enhances exosome biogenesis via AKT phosphorylation involving Rab7a and Rab11a [67].

Proteomic analyses of exosomes derived from *Mtb*-infected macrophages have identified numerous immunogenic proteins. For example, LC-MS/MS studies revealed 41 proteins in exosomes from infected macrophages and 29 proteins from culture filtrate protein (CFP)-treated macrophages, many of which are highly antigenic, including the antigen 85 complex (Rv3804c, Rv1886c, Rv0129), Mpt32, and HspX [68]. These exosomes can activate macrophages and dendritic cells, promoting T cell activation, which suggests potential applications in vaccine development. However, exosomes have also been reported to suppress IFN-γ-mediated macrophage activation, indicating a dual role in immune regulation. Notably, exosomal components such as Hsp70 and the 19 kDa lipoprotein act as key mediators of inflammation through TLR/MyD88-dependent signaling pathways [69]. Comparative proteomic analyses have also demonstrated significant alterations in exosomal protein composition following *Mtb* infection. For instance, 41 proteins were found to be significantly enriched in exosomes from infected THP-1 macrophages, many of which are membrane-associated and may play key roles in host–pathogen interactions, thus serving as potential biomarkers for tuberculosis [68]. Beyond protein cargo, exosomal non-coding RNAs regulate trained immunity. Plasma exosomal profiling of TB-resistant individuals identified the long non-coding RNA TRCR1, which interacts with FXR2 to stabilize CLOCK mRNA, promoting histone H3 (K9/K14) acetylation at immune gene promoters and establishing epigenetic memory-mediated antimicrobial activity (Table 1). *Mtb*-secreted MPT53 further stimulates lung epithelial cells to release TRCR1-enriched exosomes, enhancing host protection and improving BCG vaccine efficacy in mice [70].

Exosomes further contribute to immune modulation by delivering cytokines and chemokines and influencing macrophage and T cell polarization. Exosomes isolated from BALF have demonstrated vaccine potential, as they can induce antigen-specific IFN-γ and IL-2 production in CD4^+^ and CD8^+^ T cells and promote a Th1-skewed immune response, which is critical for protection against tuberculosis (Figure 1) [69].

Mechanistically, host ubiquitination pathways have been implicated in sorting mycobacterial proteins, such as HspX and GroES, into exosomes, suggesting that these proteins possess intrinsic signals for trafficking into MVBs during exosome biogenesis [66]. In addition to proteins, exosomes serve as carriers of functional RNA. Exosomal RNA (esRNA) derived from *Mtb*-infected macrophages includes host and pathogen transcripts, which are biologically active in recipient cells and can modulate gene expression. These RNAs influence key pathways, including MAPK signaling, TLR signaling, apoptosis, and T cell activation, and may serve as potential biomarkers for tuberculosis (Figure 1) [22].

Furthermore, studies have identified two distinct populations of extracellular vesicles released from infected macrophages: host-derived exosomes expressing markers such as CD9 and CD63, and bacterial membrane vesicles containing mycobacterial lipoglycans and lipoproteins. These bacterial vesicles can inhibit phagosome maturation, suppress MHC class II antigen presentation, and modulate TLR2-dependent cytokine responses (Figure 2) [51].

*Mtb*-derived exosomes also modulate host metabolism and inflammatory signaling. They reprogram mitochondrial respiration, regulate oxidative stress and inflammatory pathways, and contain host factors such as HIF-1α, galectins, and Hsp90 that promote HIV-1 reactivation; these effects are attenuated by N-acetyl cysteine (NAC) or inhibitors of galectins and Hsp90 [71]. The persistence of *Mtb* DNA and bacilli in mesenchymal stem cells after anti-tubercular therapy suggests that MSCs may function as a dormant bacterial reservoir [72]. In addition, exosomes released from *Mtb*-infected mesenchymal stem cells (Exo-MSCs-*Mtb*) are internalized by macrophages and induce TLR2/4-MyD88-dependent production of TNF-α, RANTES, and iNOS, eliciting robust pro-inflammatory responses both in vitro and in vivo [73]. Collectively, these findings demonstrate that *Mtb*-derived exosomes orchestrate immune activation, epigenetic reprogramming, and inflammatory signaling, underscoring their potential as therapeutic and vaccine targets. Importantly, exosomes released during *Mtb* infection exhibit dual and often opposing roles in host immunity. On one hand, they promote protective immune responses by enhancing antigen presentation, stimulating pro-inflammatory cytokine production, and activating macrophages and T lymphocytes. On the other hand, these vesicles may facilitate pathogen survival by suppressing IFN-γ-mediated macrophage activation, modulating apoptosis and autophagy pathways, and creating an immunosuppressive microenvironment that favors persistent infection [2,74]. Thus, the biological outcome of exosome signaling during tuberculosis likely depends on the composition of their cargo and the stage of infection.

**Table 1:** Biological and clinical significance of TB exosome cargos.

Exosomes	Source	Pathways Regulated	Function
miRNA			
*hsa-miR-30a-3p*, *hsa-miR-224-5p*, *hsamiR-1246*, *hsa-miR-185-5p*, *and hsa-miR-1290*	Tuberculous effusion (*Mtb*)-derived exosomal miRNA	Oxidative phosphorylation, ribosome biogenesis, mitochondrial adenosine triphosphate synthesis, and NADH dehydrogenase activity.	Indirect links between TB-derived exosomal miRNAs and lung cancer-associated genes [75]
*miR-17-5p*	Thp1 infected *M. avium*-derived exosomes and serum samples	Increased expression leads to reduced MAPK and phosphorylated ERK, JNK, and p38 pathways, which results in low TNF-a, IL-6 and IL-1b cytokines, and impaired iNOS and ROS in CFU-infected macrophages.	Involved in MAP3K2-mediated MAPK signaling [76]
*hsa-miR-122-5p_R-1*, *hsa-miR-23b-3p_R + 1*, *and hsa-miR-15a-5p_R-1*	MDR TB patients Plasma exosomes	Interacts with NTRK2, KIDINS220, NCKAP1, MAPK9, NFAT5, ATF6, KNG1, CE and SLC11A2; potentially involved in MDR-TB.	Regulation of transcription and protein binding, and MAPK signaling. Potential diagnostic value of miR-122-5p in DR TB patients [77,78]
*hsa-miR-107*	Thp1 and PBMC macrophages and Plasma TB-derived exosomes	Regulates Wnt pathways, targets Wnt16 and promotes aerophagy.	Increases ROS production and marker for TB [79]
*miR-766-3p*	Serum of active TB patients’ exosomes	Facilitates *Mtb* survival by targeting NRAMP1 (natural resistance-associated macrophage protein 1).	Indicator of TB vulnerability [80]
a. *hsa-let-7i-5p*, *hsa-miR-24-3p*, and *hsa-miR-92a-3p* b. *hsa-miR-20a-5p*, *hsa-let-7e-5p*, and *hsa-miR-26a-5p)* c. *hsa-miR-21-5p*, *hsa-miR-19a-3p*, *hsa-miR* *19b-3p*, and *hsa-miR-146a-5p*	Derived from exosome samples of HIV-TB, extra pulmonary TB and pulmonary TB of Indian cohorts	Genes are involved in MAPK, Ras and cancer signaling pathways.	TB Diagnostic marker [81]
*hsa-miR-7850-5p*, *hsa-miR-1306-5p*, *hsa-miR-363-5p*, *has-miR-374a-5p*, *hsa-miR-4654*, *has-miR-6529-5p*, *and hsa-miR-140-5p*	Plasma exosome samples of Latent *Mtb*-infected patients	Enriched genes are involved in fatty acid metabolism and ferroptosis, and MAPK signaling pathways.	Promote intracellular survival of *Mtb* in Thp1 cells and hsa-miR-7850-5p-regulated SLC11A1 gene expression [82]
*miR-20a*, *miR-20b*, *miR-26a*, *miR-106a*, *miR-191*, *and miR-486*	HIV-TB plasma samples exosomes samples	Involved in autophagy, inflammatory responses, and apoptosis.	Potential biomarker for HIV-TB coinfection [83]
*miR-125b-5p*	Spinal tuberculosis-derived exosomes	Enrichment shows involvement of MAPK, TNF, Ras, Rap1, and PI3K-Akt signaling.	Diagnostic biomarker [34]
*mmu-miR-27b-3p*, *mmu-miR-93-5p*, *mmu-miR-25-3p*, *mmu-miR-1198-5p*, *mmu-let-7c-5p, and let-7a-5p*	Raw 264.7 infected with BCG exosomes	Nucleotide metabolism, lipid metabolism, amino acid metabolism, carbohydrate metabolism, glycan biosynthesis and metabolism, and apoptosis.	Immune evasion strategy of Mycobacteria BCG infection in macrophages [84]
*miR-let-7e-5p*, *-197-3p, and -223-3p.*	MDR-TB patient-derived exosome samples.		Diagnostic biomarker [85]
*miR-185-5p*	Thp1 infected with *Mtb* H37Ra, BCG and H37Rv exosomes	Enriched genes are involved in tight junction, neuroactive ligand–receptor interaction and adipocytokine signaling pathway.	Novel TB biomarker [86]
*mmu-miR-18a*	Raw 264.7 cells infected with *Mtb* exosomes	It regulates the autophagy process by regulating *LC3*, *AMPK* and *mTOR* genes.	Upregulated during *Mtb* infection and promotes intracellular *Mtb* growth by suppressing autophagy process [87].
*TB (hsa-miR-1246*, *hsa-miR-2110*, *hsa-miR-370-3P*, *hsa-miR-28-3p*, *and* *hsa-miR-193b-5p)* *LTBI (hsa-let-7e-5p*, *hsa-let-7d-5p*, *hsa-miR-450a-5p*, *and hsa-miR-140-5p)*	Serum exosomes of active and latent TB patients.	Involved in phagocytosis, autophagy, and macrophage polarization.	Potential biomarker of active and latent TB [88]
*miR-484*, *miR-425*, and *miR-96*	Serum exosomes with active TB patients	Endocytosis, TGF-β receptor signaling pathways, N-Glycan biosynthesis, ubiquitination, proteasome degradation, and calcium signaling pathway.	Potential TB biomarker [89]
*miR-1224*, *miR-1293*, *miR-425*, *miR-4467*, *miR-4732*, *miR-484*, *miR-5094*, *miR-6848*, *miR-6849*, *miR-4488, and miR-96*	Human MDM infected with BCG-derived exosomes	Enriched in central carbon metabolism, fatty acids and sugar metabolism, amino acid metabolism, bacterial invasion-related pathways, and cell signaling pathways.	BCG-specific exosomal miRNAs [90]
*miR-155*, *miR-125b*, *and miR-29a*	Active TB samples PBMC	Controls macrophage differentiation and functionality, IFN-γ, TNF-α production.	TB biomarker [91]
Long non-coding RNA			
*Tuberculosis resister-derived CLOCK regulator 1 (TRCR1)*	TB resisters (RSTRs) individuals	It binds with RNA binding protein (FXR2) to stabilize CLOCK mRNA by forming lncRNA–mRNA complexes in monocytes and promotes H3 acetylation (K19/K14).	Exosomal TRCR1 increases anti-TB immune response and improves BCG vaccine efficacy [70]
*LOC152742*	Sputum, plasma of normal individuals, active tuberculosis patients	Transcription activation and inhibition, messenger RNA translation, organellar biogenesis, and cell development.	Biomarker for diagnosis and therapy of active tuberculosis [92].
*NONHSAT078957.2*, *NONHSAT101518.2*, *NONHSAT148822.1*, *and NONHSAT067134.2*	Plasma samples of ATB patients		TB biomarker [93]
*lncRNAs uc.48+, and NR_105053*	Plasma samples of ATB patients	Toll-like receptor signaling pathway and Jak-STAT signaling pathway.	TB biomarker [94]
Circular RNA			
*hsa_circRNA_091692*, *hsa_circRNA_102296*, *hsa_circRNA_029965*, *hsa_circRNA_103571*	Active TB patients	Autophagy, fatty acid biosynthetic process, regulation of actin cytoskeleton organization, ras signaling pathway, ubiquitin-mediated proteolysis and regulation of actin cytoskeleton, WNT signaling pathway, T-cell receptor signaling pathway, MAPK signaling pathway, B-cell receptor signaling.	Upregulated during active TB infection [95]
*hsa_circ_0000414*, *hsa_circ_0000681*, *hsa_circ_0002113*, *hsa_circ_0002362*, *hsa_circ_0002908*, *hsa_circ_0008797*, *hsa_circ_0063179*	PBMCs from patients with active tuberculosis (TB)	Cytokine–cytokine receptor interaction, Chemokine signaling pathway, neurotrophin signaling pathway, and bacterial invasion of epithelial cells.	Upregulated in active TB [96]
Proteins			
*Mtb* proteins MPT64, Ag85A, and CFP-10	Active and latent TB patient plasma exosomes	*M.tb* antigen involved in cell wall, membrane function, intermediate metabolism, respiration, and host–pathogen interactions	Diagnostic marker [97]
C1R, C1S, C2, MASP2, C4B, CFI, C9, CLU, CFHR3, SERPING1, KNG1, F5, FGA, FGG, SERPINA1, SEPRINA5, SEPRND1, and SEPRINF2	Plasma exosomes of patients with NTM, MAC and *Mtb*	Upregulated protein expression enriched in HIF-1 signaling pathway and phagosome processes and p53 signaling pathways. They are mostly involved in complement activation, coagulation and cascade protein excluding MASP2.	Diagnostic marker [38]
APOA1, APOB, and APOC1	Plasma exosomes of patients	Enriched in antioxidant activity, cyanamide hydrase activity and arylesterase activity, fibroblast migration and morphogenesis of epithelium.	Diagnostic marker [98]
C4BPA and S100A9	Plasma exosomes proteins latent tuberculosis infection	S100A9 regulates the role of CD11b, neutrophil accumulation, granuloma formation and the C4BPA canonical pathway of complement activation.	Diagnostic biomarker [37]
*Mtb* proteins RpoC, Rv2285, and HycE, apolipoproteins Host proteins Cathelicidin, SSC5D, and FCN3, SM34:1; O2 and PC 34:2.	TB lymphadenitis (TBL) plasma exosomes samples	Identified proteins and lipids are involved in and linked to immune evasion, cell identification, energy storage, and dormancy.	Cell–cell adhesion, complement activation and lipid transportation [36]
Host proteins FGBβ, HCG1745306, CRAα, δ-globin, C9, CA, EMILIN-1, LBP, TRP, A2M, TF, ORM2, and CP	Serum exosomes of ATB patients	Enriched in molecules with molecular binding and catalytic activities.	Diagnostic biomarker [99]
*Mtb* Hsp16.3, protein	U937 cells infected with *Mtb* and patient blood-derived exosomes.		TB biomarker [100]
KYAT3, SERPINA1, HP, and APOC3	Serum samples of active TB patients	Host proteins involved in neutrophil degranulation, plasma heme scavenging, and kynurenine and lipid metabolism.	Downregulated during TB infection [101]
DNA			
IS6110 and rpoB	Salivary exosomes of TB patients		Diagnostic biomarker [102,103]
MPT64, Ag85A, and CFP-10	Active and latent TB patients’ plasma exosomes		Diagnostic biomarker [97]

### 1.6. Exosome-miRNA and TB

Exosomal RNA (esRNA), including both miRNA and mRNA, represents a promising source of biomarkers in tuberculosis. These RNA molecules play a critical role in modulating the transcriptome and gene expression of recipient host cells. Although the functional role of miRNAs in mycobacterial infection is still emerging, it is evident that specific miRNAs are differentially regulated during infection, potentially conferring an advantage to the pathogen by modulating host immune responses. Both bacterial factors and host cellular mechanisms contribute to the regulation of miRNA expression in infected cells.

MicroRNAs (miRNAs) are small (20–22 nt), highly conserved non-coding RNAs that regulate gene expression at the post-transcriptional level. They are transcribed from intronic or exonic regions by RNA polymerase II/III as primary transcripts and undergo sequential processing by RNase III enzymes, Drosha and Dicer. In the nucleus, the microprocessor complex (Drosha and DGCR8) cleaves primary miRNA into ~70 nt precursor miRNA (pre-miRNA), which is exported to the cytoplasm via Exportin-5. There, Dicer further processes it into a ~20 nt miRNA duplex, from which one strand is incorporated into the RNA-induced silencing complex (RISC). The miRNA-loaded RISC binds target mRNAs, leading to translational repression or mRNA degradation [104,105,106,107].

During infection, *Mtb* modulates host miRNA expression as a strategy to manipulate immune responses and promote its survival (Table 1) [108]. Accurate normalization is critical for circulating miRNA analysis. Traditionally, RNU6B and miR-16 have been the most commonly used reference genes; however, their stability varies across biological conditions. Geometric ranking analysis identified miR-22-3p as the most stable reference gene, followed by miR-93-5p, whereas miR-16-5p showed the lowest stability. Overall stability ranked as let-7i-5p > let-7a-5p > miR-16-5p > miR-22-3p > miR-93-5p, suggesting that host and environmental factors can significantly influence circulating miRNA expression and stability [109]. Several miRNAs, including miR-146a and miR-155, are differentially regulated during *Mtb* infection [108,110,111]. Comparative analyses in macrophages infected with *Mycobacterium avium* have shown downregulation of miRNAs such as miR-20a, miR-191, miR-378, and miR-185, while miR-155, miR-146a/b, miR-886-5p, miR-29a, and let-7e are upregulated [112]. Functional studies indicate that miR-29a and let-7e target key apoptotic mediators, including caspases 3 and 7, thereby contributing to the inhibition of apoptosis during infection. Notably, miR-29a is elevated in both serum and sputum of patients with active tuberculosis, highlighting its potential as a biomarker [113]. miR-155 and miR-125b play important roles in regulating TNF-α expression during *Mtb* infection [114]. While miR-125b directly targets TNF-α mRNA, miR-155 enhances TNF-α production by targeting SHIP1, a negative regulator of the PI3K/AKT pathway [108]. However, miR-155 also promotes bacterial survival by inhibiting COX-2 and IL-6 production and activating the AKT signaling pathway [111]. Additionally, miR-155 can induce autophagy by repressing components of the mTOR pathway [108,115]. Among disease-associated miRNAs, miR-21 has emerged as a key regulator of immune responses. Persistent bacterial and viral infections induce miR-21 expression, promoting immunosuppressive and anti-inflammatory environments characterized by Th2 cells, regulatory T cells, and M2 macrophages while suppressing Th1 and M1 responses. Dysregulation of miR-21 by pathogen-associated molecular patterns (PAMPs) or damage-associated molecular patterns (DAMPs) may therefore facilitate immune evasion and disease progression [116]. In tuberculosis, mycobacteria exploit macrophages to induce miR-21 expression, thereby interfering with host immunity. Additionally, miR-21 activates inflammatory signaling in non-hematopoietic cells, contributing to tumorigenesis [117].

Other miRNAs contribute to host–pathogen interactions through diverse mechanisms. For instance, miR-142-3p regulates actin dynamics and limits phagocytosis by targeting N-WASP, while miR-21 promotes apoptosis in infected dendritic cells by targeting Bcl-2 [118]. miR-99b is upregulated in macrophages and dendritic cells during infection and suppresses immune responses; its knockdown enhances pro-inflammatory cytokine production, including IL-6, IL-12, and IL-1β [119,120], and modulates TNF-α and TNFRSF-4 expression. Furthermore, inhibition of miR-29 increases IFN-γ production in T cells during *M. bovis* infection, underscoring its role in immune regulation (Figure 1) [113,121].

Several miRNAs have also shown promise as tuberculosis biomarkers [108]. Profiling of peripheral white blood cells from children with tuberculosis identified 29 differentially expressed miRNAs, of which 14 were validated. Compared with healthy controls, children with TB exhibited increased miR-29 expression and decreased miR-155, miR-31, miR-1, miR-146a, miR-125b, miR-150, and miR-10a, highlighting their potential as early diagnostic biomarkers for pediatric tuberculosis [122]. Importantly, exosome-derived miRNAs are functionally active in recipient cells and participate in key signaling pathways, including calcium signaling, natural killer cell-mediated cytotoxicity, JAK–STAT, and MAPK pathways. Comparative profiling of exosomal miRNAs from infected versus uninfected macrophages further highlights their role in regulating host immune responses and supports their potential as diagnostic biomarkers in tuberculosis [114]. Beyond tuberculosis, exosomal miRNAs also contribute to cancer progression. Elevated levels of exosomal miR-130b-3p and miR-423-5p in pleural effusion, but not plasma, enhance p65 and Cyclin D1 expression, thereby promoting lung cancer development [123,124]. These findings underscore the diagnostic and prognostic potential of exosomal miRNAs across infectious diseases and cancer.

Exosome-associated miRNAs display both protective and pathogenic functions during tuberculosis infection. Certain miRNAs enhance host defense by promoting autophagy, inflammatory signaling, and T-cell activation, thereby contributing to bacterial clearance. Conversely, other miRNAs suppress apoptosis, inhibit pro-inflammatory cytokine production, and modulate macrophage responses in ways that facilitate immune evasion and intracellular survival of *Mtb*. These findings highlight the complex regulatory role of exosomal miRNAs in balancing host protection and disease progression.

### 1.7. Exosomes–Circular RNA and Tuberculosis

Circular RNAs (circRNAs) are a class of non-coding RNAs generated through back-splicing, in which the 3′ end of an RNA molecule is covalently linked to its 5′ end during precursor mRNA processing, forming a stable circular structure. This process is mediated by intronic complementary sequences or RNA-binding proteins (RBPs). Alternatively, circRNAs can arise via a lariat-driven mechanism, where intron-containing lariat structures formed during splicing are further processed into circular RNAs. Exosomes and other extracellular vesicles can encapsulate circRNAs and transport them to recipient cells, where they remain functionally active following uptake [125].

Emerging evidence highlights circular RNAs (circRNAs) as important regulators of host immune responses during tuberculosis (TB) infection (Table 1) [126]. circRNAs modulate key host defense mechanisms, including monocyte survival, autophagy, and macrophage polarization, thereby influencing resistance to *Mtb* [127,128]. Owing to their high stability, abundance in body fluids, and resistance to degradation, circRNAs have also emerged as promising non-invasive biomarkers for TB diagnosis [129,130]. Recent studies have highlighted the importance of circRNAs in *Mtb* infection, where they regulate key biological processes such as apoptosis, autophagy, and macrophage polarization, thereby influencing host immune responses [131]. Due to their structural stability and disease-specific expression patterns, circRNAs have emerged as promising biomarkers for tuberculosis diagnosis, prognosis, and treatment monitoring.

In active tuberculosis, several circRNAs are upregulated, including hsa_circ_103571 in plasma; hsa_circ_0043497 and hsa_circ_0001204 in infected human monocyte-derived macrophages (MDMs); and circRNA_051239, circRNA_029965, and circRNA_404022 in serum samples (68, 69, 70). Conversely, circRNAs such as hsa_circ_0005836 and hsa_circ_0009128 are downregulated in peripheral blood mononuclear cells (PBMCs) of patients with active disease. Some circRNAs also exhibit dynamic changes during therapy; for example, hsa_circRNA_001937 is upregulated during anti-tuberculosis treatment and decreases following therapy, indicating its potential as a treatment-response biomarker. Additional circRNAs, including hsa_circ_0001204, hsa_circ_0001747, hsa_circ_0001953, and hsa_circ_0009024, show differential expression patterns associated with disease progression.

Functionally, circRNAs participate in key signaling pathways relevant to tuberculosis pathogenesis. For instance, circRNA_101128 modulates the MAPK and PI3K–Akt pathways via let-7a and exhibits an inverse correlation with its expression in TB patients. circRNA_051239 is elevated in drug-resistant tuberculosis, suggesting a role in disease severity. Certain circRNAs also regulate autophagy, a critical host defense mechanism; for example, circAGFG1 promotes autophagy via the miR-1257/Notch axis, while hsa_circ_0045474 modulates macrophage autophagy through the miR-582-5p/TNKS2 axis. In addition, circRNAs influence macrophage polarization and immune regulation during infection. hsa_circ_0003528, which regulates CTLA4 expression, is upregulated in active tuberculosis and contributes to immune modulation [129]. hsa_circ_0002371 is significantly upregulated in TB patients and promotes hsa-miR-502-5p, which suppresses the autophagy-related gene ATG16L1, thereby inhibiting macrophage autophagy and facilitating intracellular *Mtb* survival [132]. Conversely, downregulation of hsa_circ_0045474 enhances macrophage autophagy through the miR-582-5p/TNKS2 signaling pathway [133]. circRNA-0003528 regulates macrophage polarization by suppressing miR-224-5p, miR-324-5p, and miR-488-5p, while increasing CTLA4 expression [134]. In active TB, circAGFG1 promotes autophagy and inhibits apoptosis via the miR-1257/Notch pathway [131]. In contrast, circRNA_SLC8A1 enhances intracellular *Mtb* survival by upregulating the autophagy-related protein SQSTM1/p62 and activating the NF-κB signaling pathway [108,135]. Collectively, these findings demonstrate that circRNAs play dual roles in regulating autophagy, apoptosis, and macrophage function, making them attractive candidates as both diagnostic biomarkers and therapeutic targets in tuberculosis.

Similar to miRNAs, exosomal circRNAs exert dual immunomodulatory effects during *Mtb* infection. Some circRNAs contribute to host protective responses by regulating autophagy, macrophage activation, and inflammatory signaling pathways involved in bacterial clearance (Table 1). In contrast, other circRNAs may support pathogen persistence by altering macrophage polarization, suppressing immune responses, and promoting intracellular survival of mycobacteria. Therefore, circRNAs may act as critical regulators of the host–pathogen interaction during tuberculosis.

### 1.8. Diagnostic and Prognostic Potential of Exosomes in Tuberculosis

Exosomes represent a promising source of blood-based biomarkers for tuberculosis (TB). Their presence in readily accessible body fluids enables non-invasive sampling, making them attractive candidates for diagnostic applications.

As discussed in the previous section, numerous miRNAs and exosomal circRNAs exhibit dual immunomodulatory effects during *Mtb* infection. Both are attractive candidates as TB biomarkers because their incorporation into exosomes protects them from extracellular degradation, allowing relatively stable detection in body fluids. miRNAs are short, abundant, and extensively studied, with well-established roles in post-transcriptional gene regulation and several reported TB-associated signatures [109]. However, their broad target spectrum, tissue- and cell-type specificity, and variability in normalization and exosome isolation methods may limit diagnostic reproducibility. In contrast, circRNAs are covalently closed RNA molecules lacking free 5′ and 3′ ends, making them highly resistant to exonuclease-mediated degradation and potentially more stable than linear RNAs. Their abundance and disease-associated expression patterns make them promising biomarkers; however, circRNA research in TB remains relatively limited, with gaps in understanding their biogenesis, functional mechanisms, standardized detection, and clinical validation [126]. Thus, miRNAs currently have a stronger evidence base for clinical application, whereas circRNAs may offer advantages in stability and biomarker specificity but require larger multicenter cohorts and standardized methodologies before clinical translation.

Proteomic analyses have identified distinct exosomal protein signatures associated with tuberculosis (TB), highlighting their diagnostic and prognostic potential. Serum exosomes from patients with active TB (ATB) contained 123 differentially expressed proteins, including increased lipopolysaccharide-binding protein (LBP) and decreased CD36 and MHC-I, all of which showed diagnostic potential by ROC analysis [99]. In latent TB infection (LTBI), exosomes contained *Mtb*-derived peptides involved in nitrogen metabolism, particularly GarA and glutamine synthetase (GlnA1), as well as heat-shock proteins GroES and DnaK. Notably, a GlnA1 peptide was detected in 82% of LTBI patients, suggesting its utility as a pathogen-specific biomarker [136]. Furthermore, Hsp16.3 was detected exclusively in plasma exosomes from ATB patients, enabling differentiation from LTBI [100]. Exosomal protein profiles also distinguish pulmonary TB from TB lymphadenitis and monitor treatment responses. Plasma exosomes from TB patients were enriched in haptoglobin (HP), proteoglycan-4, CD151, stomatin, ICAM-2, α1-acid glycoprotein-1, SLC2A3, and serum amyloid A1, whereas immunoglobulins, GRIP1, and complement C1r were enriched in TB lymphadenitis [36]. Following anti-TB therapy, S100A9 and C4BPA decreased in LTBI patients, while proteins including myosin-9, IGHV4-28, and GRIP1 increased and HP, ficolin-3, SAA4, and apolipoprotein B-100 decreased in treated TB patients, indicating their potential as prognostic biomarkers [36,37].

Exosomal lipid profiling further reflects TB pathogenesis. Plasma exosomes from TB patients are enriched in triacylglycerols and cholesteryl esters (CEs), lipids that support *Mtb* survival and dissemination [36]. Additionally, *Mtb* produces immunomodulatory glycolipids, including trehalose-6,6′-dimycolate, lipomannan, lipoarabinomannan (LAM), and phosphatidylinositol mannosides (PIMs), which are recognized by pattern recognition receptors (PRRs) and modulate innate immune responses while contributing to phagosome maturation arrest [137,138]. We recently developed a cubosome-based lipid nanocarrier encapsulating the mycobacterial cord factor trehalose 6,6′-dimycolate (TDM) to overcome its poor solubility and toxicity. The formulation enhanced macrophage activation, epigenetically reprogrammed pro-inflammatory responses, improved antigen presentation to CD4^+^ T cells, and promoted protective anti-*Mtb* immunity, highlighting its potential as a novel TB nanovaccine [64].

Exosomal cargo, including proteins and RNA, plays a critical role in modulating gene expression in recipient cells. Comprehensive proteomic and transcriptomic analyses of exosomes derived from *Mtb*-infected cells may reveal candidate molecules involved in host immune modulation. Such insights could facilitate the identification of novel diagnostic markers as well as therapeutic targets, ultimately contributing to improved management of tuberculosis. Recent small-scale clinical and pilot studies have highlighted the promising potential of exosomes as biomarkers of *Mtb* infection. We summarize the current evidence for the sensitivity, specificity, and validation status of key exosomal biomarkers for which such data are available (Table 2). However, many candidate biomarkers remain insufficiently validated, with limited clinical cohorts and diagnostic performance data. Larger, well-designed, and independently validated clinical studies are therefore needed to establish their diagnostic utility and clinical applicability.

The dual role of *Mtb*-derived exosomes further enhances their significance as potential biomarkers. Exosomes carrying pro-inflammatory and immune-activating molecules may reflect protective host responses, whereas exosomes enriched with immunosuppressive factors may indicate active disease progression or pathogen persistence. Consequently, profiling exosomal cargo could not only improve TB diagnosis but also provide insights into disease stage, severity, and treatment response.

## 2. Future Prospectives

Exosomes have emerged as critical mediators of host–pathogen interactions during *Mtb* infection. By transporting proteins, lipids, DNA, mRNAs, miRNAs, lncRNAs, and circRNAs, they enable communication between infected and uninfected cells, thereby shaping immune responses, antigen presentation, inflammation, tissue remodeling, and disease progression (Table 1). Depending on their cellular origin and molecular cargo, exosomes can either enhance protective immunity or promote immune evasion and bacterial persistence, emphasizing their dual role in tuberculosis (TB).

Despite significant advances, several challenges must be overcome before exosome-based technologies can be translated into clinical practice. A major limitation is the lack of standardized methods for exosome isolation, purification, characterization, and quality control. Differences in isolation techniques, culture conditions, and analytical platforms contribute to batch-to-batch variability and poor reproducibility, making it difficult to compare studies and identify robust biomarkers. Future efforts should focus on developing scalable manufacturing processes, reducing the heterogeneity of both native and engineered exosomes, determining whether size- or cargo-based sorting can improve functional consistency, and establishing standardized quality-control criteria for clinical applications.

A comprehensive understanding of exosome biology throughout the course of TB infection is also needed. Future studies should define the temporal changes in exosomal cargo, identify the cellular sources and recipient cells, and elucidate the signaling pathways governing exosome biogenesis, cargo loading, uptake, and downstream immune modulation. Selective modulation of exosome biogenesis or release may represent a novel host-directed therapeutic strategy and help clarify whether exosomes predominantly benefit the host or are exploited by *Mtb* to evade immune surveillance.

The diagnostic and therapeutic potential of exosomes also warrants further validation. The stability of exosomal nucleic acids makes them attractive biomarkers; however, most studies evaluating miRNAs, lncRNAs, circRNAs, proteins, and lipids have been limited by small cohorts, heterogeneous methodologies, and inconsistent validation. Large, multicenter studies using standardized protocols are needed to establish clinically reliable biomarker panels for distinguishing active TB, latent TB infection, treatment response, and disease prognosis. Importantly, exosomal biomarkers should currently be viewed as complementary tools rather than replacements for established diagnostic methods.

Exosomes are most extensively studied in the context of infection biology and tumor immunology. Because of their small size, they can easily cross the blood–brain barrier and may contribute to the spread of pathogenic factors, transmission of prion diseases, or the induction of pro-inflammatory conditions [35,143]. Therefore, the effects of *Mtb*-derived exosomes may extend beyond tuberculosis, influencing other pathological conditions through their interactions with diverse tissues and organ systems. For example, the remarkable ability of exosomes to cross biological barriers, including the blood–brain barrier, further expands their therapeutic potential as well as detrimental role in brain health. Interestingly, the effects of BCG immunotherapy may extend well beyond tuberculosis. Several epidemiological studies have reported that bladder cancer patients treated with intravesical BCG have a significantly lower risk of developing Alzheimer’s disease, Parkinson’s disease, and other dementias [144,145,146]. Experimental studies further show that BCG can reduce amyloid-β pathology, dampen neuroinflammation, preserve synaptic integrity, and improve cognitive function in animal models [147,148,149]. More recently, two open-label clinical trials demonstrated that BCG immunotherapy induces trained immunity-like changes in the central nervous system and alters Alzheimer’s-related biomarkers, including changes in cerebrospinal fluid amyloid-β levels, suggesting that BCG can remodel CNS immune responses in humans [150]. While mesenchymal stem cell (MSC)-derived exosomes have shown promising neuroprotective effects in experimental models of Alzheimer’s disease and Parkinson’s disease through delivery of therapeutic cargo such as miR-188-3p [151,152], the neurological consequences of *Mtb*- or BCG-derived exosomes remain largely unexplored. Together, these findings raise the intriguing possibility that BCG-mediated immune reprogramming and potentially BCG-derived exosomes may influence pathways involved in neurodegeneration, although the underlying mechanisms remain to be established and require validation in larger controlled studies. Epidemiological studies suggesting neuroprotective effects of BCG vaccination raise intriguing questions regarding whether mycobacterial exosomes contribute to these observations. Exploring the role of *Mtb*- and BCG-derived exosomes in neuroinflammation and neurodegenerative diseases represents an important avenue for future investigation.

Finally, exosomes represent an attractive platform for next-generation TB vaccines, host-directed therapies, and targeted drug delivery because of their natural biocompatibility, low immunogenicity, and ability to transport bioactive cargo across physiological barriers. Although these applications remain largely preclinical, advances in exosome engineering may enable the development of safer and more effective immunotherapies and precision drug delivery systems. A deeper understanding of the molecular determinants governing the protective versus pathogenic functions of exosomes will be essential for translating these promising vesicles into clinically meaningful tools for tuberculosis prevention, diagnosis, and treatment.

Exosomes (50–150 nm) released from *Mtb*-infected macrophages carry host- and pathogen-derived cargo, including miRNAs, proteins, lipids, and antigenic components. These vesicles are internalized by recipient cells via phagocytosis, endocytosis, or receptor-mediated uptake. Following internalization, exosomal cargo activates signaling pathways through pattern recognition receptors (e.g., TLRs), leading to MyD88-dependent activation of NF-κB and modulation of cytokine expression. This results in either immune activation or suppression, depending on the cargo and cellular context. Exosomes also induce epigenetic remodeling by delivering molecules that regulate histone-modifying enzymes. Additionally, exosomal miRNAs mediate post-transcriptional gene silencing in recipient cells. Overall, exosomes from *Mtb*-infected macrophages reprogram bystander cells and may serve as potential biomarkers for tuberculosis.

## Figures and Tables

**Table 2:** Diagnostic performance of selected exosomal proteins, lipids, and RNAs in TB.

Exosomal Cargo	Clinical Relevance and Diagnostic Performance	Validation Status
Mtb-encoded small RNA (ASdes and miR5)	Active pulmonary TB; sensitivity 94.5%; specificity 100.0%	Droplet digital PCR-based single clinical study (*n* = 174); requires external validation [139].
Mtb DNA IS6110	Pulmonary TB; sensitivity 76.9% (95% CI: 60.7–88.9%); specificity 98.0% (95% CI: 94.3–99.6%) by ddPCR versus culture	Promising clinical study (*n* = 190 respiratory samples); requires independent and multicenter validation [103].
miR-20a, miR-20b, miR-26a, miR-106a, miR-191, and miR-486	Pulmonary TB and tuberculous meningitis; AUC 0.97 (95% CI 0.80–0.99) in TBM and 0.97 (0.87–0.99) in PTB	Testing cohort (*n* = 370) demonstrated good performance; requires larger external validation [140].
miR-96, miR-425, and miR-484	Active pulmonary TB; (AUC) 0.62 for miR-96 (*p* < 0.05), 0.66 for miR-425 (*p* < 0.05), and 0.72 for miR-484 (*p* < 0.05)	Small-scale pilot study (*n*= 25) [89].
LBP, CD36, and MHC-1	LBP (AUC 0.677), CD36 (AUC 0.605) and MHC-1	Exploratory; no independent validation reported [99].
C4BPA and S100A9	LTBI treatment response; AUC 0.78; sensitivity 95%, specificity 60%	Screening + validation cohorts (*n* = 100); requires external validation [37].
Urine-derived extracellular vesicles	TB vs LTBI AUC 0.952; sensitivity 88.9%, specificity 100%; TB vs controls AUC 0.852–0.857; sensitivity 88.9%, specificity 88.9–92.9%	Relatively small clinical study (*n* = 41); needs independent validation [141].
Exosomal lipid profile (SM, PC, PI, TAG, CE)	Disease-state characterization; sensitivity/specificity not established	Discovery-stage lipidomics; no clinical validation [36].
Mtb-derived LAM	Pediatric HIV-TB coinfection; sensitivity 74% overall; detected 73% of microbiologically missed cases	Clinical cohort (147 children with HIV); promising but requires broader validation [142].

## Data Availability

The original contributions presented in this study are included in the article. Further inquiries can be directed to the corresponding author.
